# Crystal structure of bis­(azido-κ*N*)bis­[2,5-bis­(pyridin-2-yl)-1,3,4-thia­diazole-κ^2^ *N* ^2^,*N* ^3^]nickel(II)

**DOI:** 10.1107/S2056989015000201

**Published:** 2015-01-14

**Authors:** Abdelhakim Laachir, Fouad Bentiss, Salaheddine Guesmi, Mohamed Saadi, Lahcen El Ammari

**Affiliations:** a Laboratoire de Chimie de Coordination et d’Analytique (LCCA), Faculté des Sciences, Université Chouaib Doukkali, BP 20, M-24000 El Jadida, Morocco; b Laboratoire de Catalyse et de Corrosion de Matériaux (LCCM), Faculté des Sciences, Université Chouaib Doukkali, BP 20, M-24000 El Jadida, Morocco; c Laboratoire de Chimie du Solide Appliquée, Faculté des Sciences, Université Mohammed V, Avenue Ibn Battouta, BP 1014, Rabat, Morocco

**Keywords:** crystal structure, mononuclear nickel(II) complex, 1,3,4-thia­diazole, azide ligand, π–π inter­actions

## Abstract

Reaction of 2,5-bis­(pyridin-2-yl)-1,3,4-thia­diazole and sodium azide with nickel(II) triflate yielded the mononuclear title complex, [Ni(N_3_)_2_(C_12_H_8_N_4_S)_2_]. The Ni^II^ ion is located on a centre of symmetry and is octa­hedrally coordinated by four N atoms of the two bidentate heterocyclic ligands in the equatorial plane. The axial positions are occupied by the N atoms of two almost linear azide ions [N—N—N = 178.8 (2)°]. The thia­diazole and pyridine rings of the heterocyclic ligand are almost coplanar, with a maximum deviation from the mean plane of 0.0802 (9) Å. The cohesion of the crystal structure is ensured by π–π inter­actions between parallel pyridine rings of neighbouring mol­ecules [centroid-to-centroid distance = 3.6413 (14) Å], leading to a layered arrangement of the mol­ecules parallel to (001).

## Related literature   

2,5-Bis(pyridin-2-yl)-1,3,4-thia­diazole has been used as a bidentate or tetra­dentate ligand forming mononuclear (Bentiss *et al.*, 2004 ▸, 2011*a* ▸, 2012 ▸; Zheng *et al.*, 2006 ▸) or dinuclear complexes (Laachir *et al.*, 2013 ▸). Coordination of the azide ion to transition metals results in compounds with inter­esting magnetic properties (Machura *et al.*, 2011 ▸; Świtlicka-Olszewska *et al.*, 2014 ▸). The iron salt with the same heterocyclic ligand and thio­cyanate as the pseudohalide was reported by Klingele *et al.* (2010 ▸). For the crystal structure of the related tetra­fluorido­borate salt of [Ni(C_12_H_8_N_4_S)_2_(H_2_O)_2_], see: Bentiss *et al.* (2011*b* ▸). For the synthesis of the heterocyclic ligand, see: Lebrini *et al.* (2005 ▸).
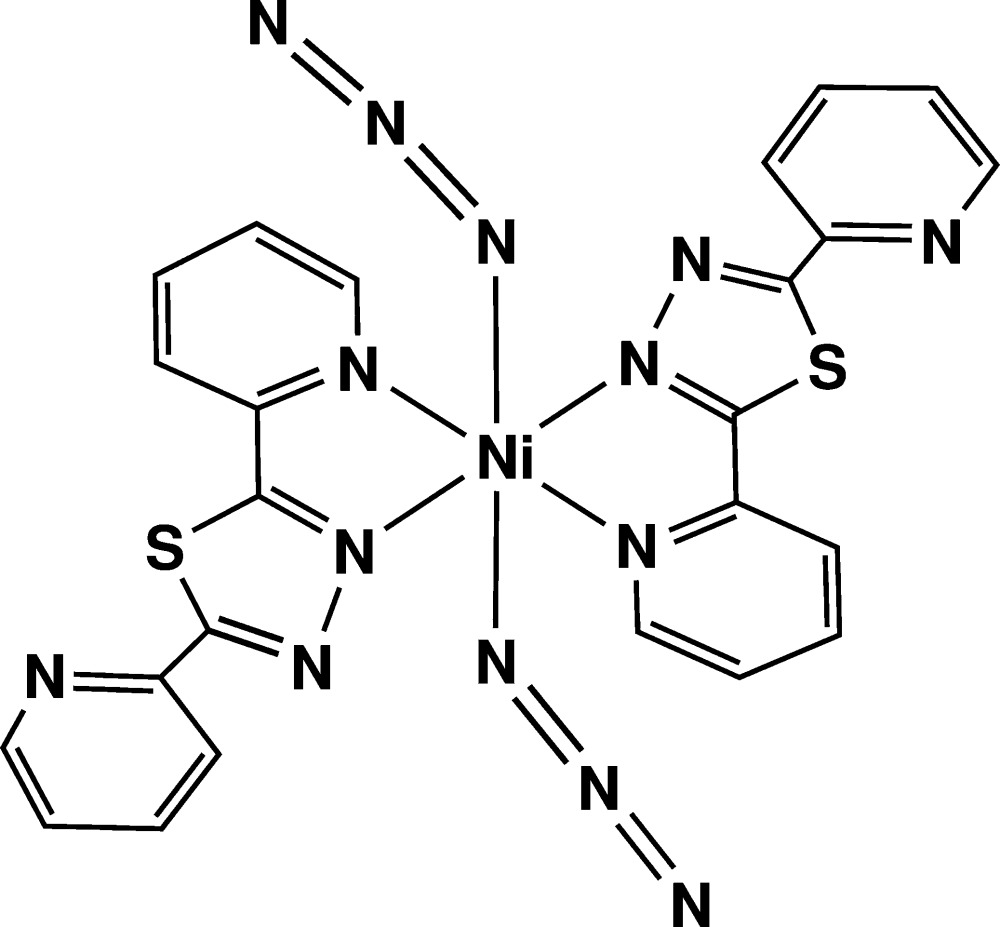


## Experimental   

### Crystal data  

[Ni(N_3_)_2_(C_12_H_8_N_4_S)_2_]
*M* *_r_* = 623.34
Monoclinic, 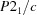

*a* = 7.7981 (3) Å
*b* = 8.2410 (3) Å
*c* = 20.1555 (7) Å
β = 93.141 (2)°
*V* = 1293.33 (8) Å^3^
*Z* = 2
Mo *K*α radiation
μ = 0.96 mm^−1^
*T* = 296 K
0.39 × 0.31 × 0.18 mm

### Data collection  

Bruker APEXII CCD diffractometer
Absorption correction: multi-scan (*SADABS*; Bruker, 2009 ▸) *T*_min_ = 0.640, *T*_max_ = 0.747
15710 measured reflections
3077 independent reflections
2643 reflections with *I* > 2σ(*I*)
*R*_int_ = 0.033

### Refinement  

*R*[*F* ^2^ > 2σ(*F* ^2^)] = 0.036
*wR*(*F* ^2^) = 0.100
*S* = 1.04
3077 reflections
187 parameters
H-atom parameters constrained
Δρ_max_ = 1.25 e Å^−3^
Δρ_min_ = −0.35 e Å^−3^

### 

Data collection: *APEX2* (Bruker, 2009 ▸); cell refinement: *SAINT* (Bruker, 2009 ▸); data reduction: *SAINT*; program(s) used to solve structure: *SHELXS97* (Sheldrick, 2008 ▸); program(s) used to refine structure: *SHELXL97* (Sheldrick, 2008 ▸); molecular graphics: *ORTEP-3 for Windows* (Farrugia, 2012 ▸); software used to prepare material for publication: *WinGX* (Farrugia, 2012 ▸).

## Supplementary Material

Crystal structure: contains datablock(s) I. DOI: 10.1107/S2056989015000201/wm5108sup1.cif

**Fig. 1.**
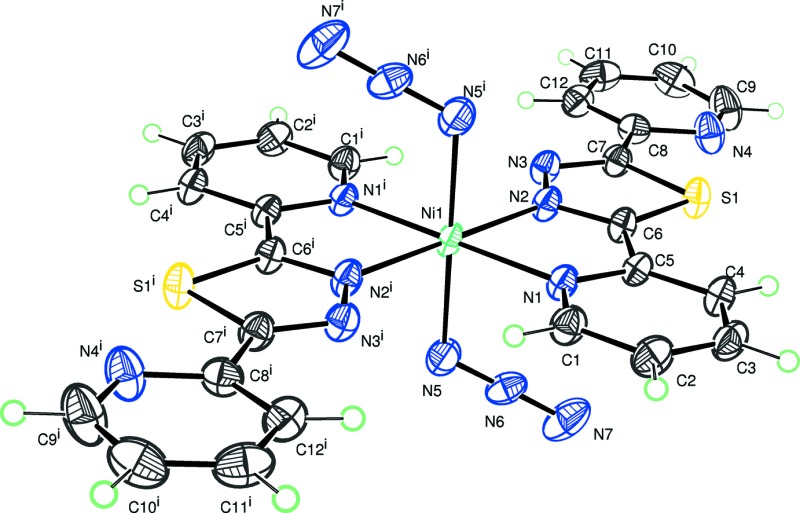
The molecular structure of the title compound. Displacement ellipsoids are drawn at the 50% probability level. H atoms are represented as spheres of arbitrary radius. [Symmetry code: (i) -x + 2, -y + 1, -z + 2.]

Structure factors: contains datablock(s) I. DOI: 10.1107/S2056989015000201/wm5108Isup2.hkl

x y z . DOI: 10.1107/S2056989015000201/wm5108fig1.tif
The mol­ecular structure of the title compound. Displacement ellipsoids are drawn at the 50% probability level. H atoms are represented as spheres of arbitrary radius. [Symmetry code: (i) −*x* + 2, −*y* + 1, −*z* + 2.]

**Fig. 2.**
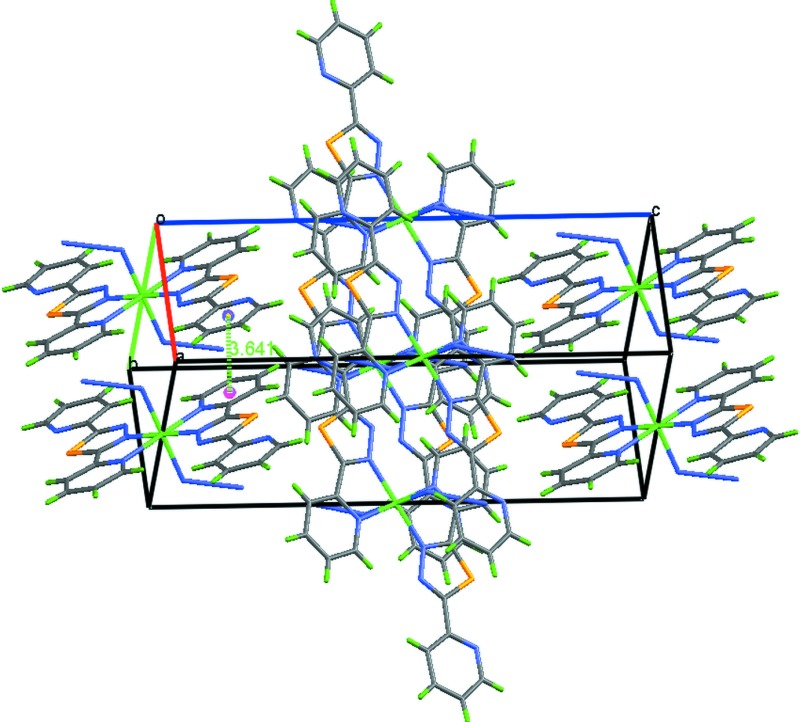
The crystal packing of the title compound, showing intermolecular π–π interactions between pyridyl rings (dashed green lines).

. DOI: 10.1107/S2056989015000201/wm5108fig2.tif
The crystal packing of the title compound, showing inter­molecular π–π inter­actions between pyridyl rings (dashed green lines).

CCDC reference: 1042351

Additional supporting information:  crystallographic information; 3D view; checkCIF report

## supplementary crystallographic information

### S1. Experimental

The 2,5-bis(2-pyridyl)-1,3,4-thiadiazole ligand (noted *L*) was synthesized as described previously by Lebrini *et al.* (2005). Ni_2_*L*_2_(N_3_)_2_ was obtained in bulk quantity by dropwise addition of an aqueous solution of NaN_3_ (0.4 mmol, 26 mg) to an ethanol/water solution of *L* (0.1 mmol, 24 mg) and Ni(O_3_SCF_3_)_2_ (0.1 mmol, 36 mg) under constant stirring at room temperature. An orange coloured solid was precipitated, filtered and washed with cold ethanol. Single crystals of Ni_2_*L*_2_(N_3_)_2_ were grown by slow interdiffusion of a solution of Ni(O_3_SCF_3_)_2_ and *L* in acetonitrile into NaN_3_ dissolved in water. Orange block-shaped single crystals appeared after one month. The crystals were washed with water and dried under vacuum (yield 50%).

CAUTION. Azide compounds are potentially explosive. Only a small amount of material should be prepared and handled with care.

### S2. Refinement

H atoms were located in a difference map and treated as riding with C—H = 0.96 Å and with *U*_iso_(H) = 1.2*U*_eq_(C). The highest electron density was found 1.65 Å from atom H1. The vicinity of this peak to the H1 atom and the requirement for electroneutrality made it seem possible that this electron density might be associated with an underoccupied water molecule. However, the *U*_eq_ value of the so modelled O atom (occupancy < 0.05) refined to negative values and hence this electron density was not considered in the final model.

### Crystal data

**Table:**

[Ni(N_3_)_2_(C_12_H_8_N_4_S)_2_]	*F*(000) = 636
*M**_r_* = 623.34	*D*_x_ = 1.601 Mg m^−^^3^
Monoclinic, *P*2_1_/*c*	Mo *K*α radiation, λ = 0.71073 Å
Hall symbol: -P 2ybc	Cell parameters from 3077 reflections
*a* = 7.7981 (3) Å	θ = 2.6–27.9°
*b* = 8.2410 (3) Å	µ = 0.96 mm^−^^1^
*c* = 20.1555 (7) Å	*T* = 296 K
β = 93.141 (2)°	Block, orange
*V* = 1293.33 (8) Å^3^	0.39 × 0.31 × 0.18 mm
*Z* = 2	

### Data collection

**Table:**

Bruker APEXII CCD diffractometer	3077 independent reflections
Radiation source: fine-focus sealed tube	2643 reflections with *I* > 2σ(*I*)
Graphite monochromator	*R*_int_ = 0.033
φ and ω scans	θ_max_ = 27.9°, θ_min_ = 2.6°
Absorption correction: multi-scan (*SADABS*; Bruker, 2009)	*h* = −10→9
*T*_min_ = 0.640, *T*_max_ = 0.747	*k* = −10→10
15710 measured reflections	*l* = −26→26

### Refinement

**Table:**

Refinement on *F*^2^	Primary atom site location: structure-invariant direct methods
Least-squares matrix: full	Secondary atom site location: difference Fourier map
*R*[*F*^2^ > 2σ(*F*^2^)] = 0.036	Hydrogen site location: inferred from neighbouring sites
*wR*(*F*^2^) = 0.100	H-atom parameters constrained
*S* = 1.04	*w* = 1/[σ^2^(*F*_o_^2^) + (0.0522*P*)^2^ + 0.8063*P*] where *P* = (*F*_o_^2^ + 2*F*_c_^2^)/3
3077 reflections	(Δ/σ)_max_ < 0.001
187 parameters	Δρ_max_ = 1.25 e Å^−^^3^
0 restraints	Δρ_min_ = −0.35 e Å^−^^3^

### Special details

**Table:**

Geometry. All e.s.d.'s (except the e.s.d. in the dihedral angle between two l.s. planes) are estimated using the full covariance matrix. The cell e.s.d.'s are taken into account individually in the estimation of e.s.d.'s in distances, angles and torsion angles; correlations between e.s.d.'s in cell parameters are only used when they are defined by crystal symmetry. An approximate (isotropic) treatment of cell e.s.d.'s is used for estimating e.s.d.'s involving l.s. planes.
Refinement. Refinement of *F*^2^ against all reflections. The weighted *R*-factor *wR* and goodness of fit *S* are based on *F*^2^, conventional *R*-factors *R* are based on *F*, with *F* set to zero for negative *F*^2^. The threshold expression of *F*^2^ > 2σ(*F*^2^) is used only for calculating *R*-factors(gt) *etc*. and is not relevant to the choice of reflections for refinement. *R*-factors based on *F*^2^ are statistically about twice as large as those based on *F*, and *R*- factors based on all data will be even larger.

### Fractional atomic coordinates and isotropic or equivalent isotropic displacement parameters (Å^2^)

**Table:**

	*x*	*y*	*z*	*U*_iso_*/*U*_eq_	
C1	1.3016 (3)	0.4676 (3)	0.90640 (11)	0.0356 (5)	
H1	1.3538	0.4022	0.9392	0.043*	
C2	1.3853 (3)	0.4935 (3)	0.84813 (12)	0.0397 (5)	
H2	1.4916	0.4460	0.8423	0.048*	
C3	1.3091 (3)	0.5902 (3)	0.79925 (10)	0.0380 (5)	
H3	1.3630	0.6089	0.7599	0.046*	
C4	1.1511 (3)	0.6589 (3)	0.80960 (10)	0.0350 (4)	
H4	1.0966	0.7246	0.7774	0.042*	
C5	1.0759 (2)	0.6281 (2)	0.86873 (9)	0.0292 (4)	
C6	0.9114 (3)	0.6956 (3)	0.88563 (9)	0.0304 (4)	
C7	0.6422 (2)	0.8193 (2)	0.90011 (10)	0.0313 (4)	
C8	0.4793 (3)	0.9088 (3)	0.89219 (11)	0.0336 (4)	
C9	0.3101 (4)	1.0642 (4)	0.82288 (15)	0.0575 (7)	
H9	0.2915	1.1167	0.7823	0.069*	
C10	0.1844 (3)	1.0747 (3)	0.86751 (15)	0.0536 (7)	
H10	0.0843	1.1332	0.8575	0.064*	
C11	0.2099 (3)	0.9966 (3)	0.92746 (14)	0.0498 (6)	
H11	0.1274	1.0021	0.9590	0.060*	
C12	0.3601 (3)	0.9095 (3)	0.94026 (11)	0.0401 (5)	
H12	0.3799	0.8534	0.9800	0.048*	
N1	1.1488 (2)	0.5333 (2)	0.91692 (8)	0.0298 (4)	
N2	0.8473 (2)	0.6603 (2)	0.94235 (8)	0.0313 (4)	
N3	0.6911 (2)	0.7303 (2)	0.95059 (8)	0.0320 (4)	
N4	0.4563 (3)	0.9844 (2)	0.83359 (11)	0.0463 (5)	
N5	0.8723 (3)	0.3030 (2)	0.95247 (9)	0.0427 (4)	
N6	0.8332 (2)	0.3118 (2)	0.89483 (9)	0.0385 (4)	
N7	0.7973 (3)	0.3188 (3)	0.83827 (11)	0.0645 (7)	
S1	0.78523 (7)	0.82456 (7)	0.83744 (3)	0.03745 (15)	
Ni1	1.0000	0.5000	1.0000	0.02650 (12)	

### Atomic displacement parameters (Å^2^)

**Table:**

	*U*^11^	*U*^22^	*U*^33^	*U*^12^	*U*^13^	*U*^23^
C1	0.0333 (11)	0.0420 (11)	0.0319 (10)	0.0063 (9)	0.0056 (8)	0.0008 (9)
C2	0.0340 (11)	0.0477 (13)	0.0387 (12)	0.0036 (9)	0.0120 (9)	−0.0044 (9)
C3	0.0386 (11)	0.0478 (13)	0.0288 (10)	−0.0037 (10)	0.0128 (8)	−0.0036 (9)
C4	0.0374 (11)	0.0451 (12)	0.0228 (9)	0.0007 (9)	0.0050 (8)	0.0023 (8)
C5	0.0301 (9)	0.0351 (10)	0.0226 (9)	0.0005 (8)	0.0046 (7)	−0.0011 (7)
C6	0.0309 (9)	0.0385 (11)	0.0219 (9)	0.0017 (8)	0.0008 (7)	0.0026 (8)
C7	0.0290 (9)	0.0367 (10)	0.0283 (9)	0.0022 (8)	0.0027 (7)	0.0006 (8)
C8	0.0291 (10)	0.0329 (10)	0.0385 (11)	0.0033 (8)	0.0006 (8)	−0.0013 (8)
C9	0.0508 (15)	0.0563 (16)	0.0656 (17)	0.0155 (13)	0.0046 (13)	0.0247 (14)
C10	0.0362 (12)	0.0439 (14)	0.081 (2)	0.0158 (11)	0.0015 (12)	−0.0001 (13)
C11	0.0355 (12)	0.0543 (15)	0.0608 (16)	−0.0010 (10)	0.0129 (11)	−0.0183 (12)
C12	0.0398 (12)	0.0451 (13)	0.0354 (11)	−0.0025 (10)	0.0021 (9)	−0.0026 (9)
N1	0.0312 (8)	0.0357 (9)	0.0229 (8)	0.0022 (7)	0.0045 (6)	0.0003 (6)
N2	0.0298 (8)	0.0410 (9)	0.0235 (8)	0.0063 (7)	0.0045 (6)	0.0019 (7)
N3	0.0283 (8)	0.0407 (9)	0.0271 (8)	0.0072 (7)	0.0029 (6)	0.0014 (7)
N4	0.0380 (10)	0.0508 (12)	0.0507 (12)	0.0098 (9)	0.0090 (9)	0.0182 (9)
N5	0.0501 (11)	0.0467 (11)	0.0315 (9)	−0.0058 (9)	0.0040 (8)	−0.0017 (8)
N6	0.0304 (9)	0.0464 (11)	0.0387 (10)	0.0060 (8)	0.0015 (7)	−0.0129 (8)
N7	0.0631 (15)	0.0907 (19)	0.0379 (12)	0.0154 (13)	−0.0121 (10)	−0.0185 (12)
S1	0.0343 (3)	0.0496 (3)	0.0289 (3)	0.0091 (2)	0.00513 (19)	0.0117 (2)
Ni1	0.02602 (19)	0.0358 (2)	0.01789 (17)	0.00586 (14)	0.00320 (12)	0.00259 (13)

### Geometric parameters (Å, º)

**Table:**

C1—N1	1.336 (3)	C9—N4	1.324 (3)
C1—C2	1.391 (3)	C9—C10	1.369 (4)
C1—H1	0.9300	C9—H9	0.9300
C2—C3	1.376 (3)	C10—C11	1.374 (4)
C2—H2	0.9300	C10—H10	0.9300
C3—C4	1.382 (3)	C11—C12	1.386 (3)
C3—H3	0.9300	C11—H11	0.9300
C4—C5	1.380 (3)	C12—H12	0.9300
C4—H4	0.9300	N1—Ni1	2.1069 (17)
C5—N1	1.348 (2)	N2—N3	1.366 (2)
C5—C6	1.456 (3)	N2—Ni1	2.0885 (16)
C6—N2	1.305 (3)	N5—N6	1.187 (3)
C6—S1	1.714 (2)	N5—Ni1	2.1075 (19)
C7—N3	1.295 (2)	N6—N7	1.160 (3)
C7—C8	1.470 (3)	Ni1—N2^i^	2.0885 (16)
C7—S1	1.731 (2)	Ni1—N1^i^	2.1069 (17)
C8—N4	1.339 (3)	Ni1—N5^i^	2.108 (2)
C8—C12	1.379 (3)		
			
N1—C1—C2	122.4 (2)	C10—C11—H11	120.5
N1—C1—H1	118.8	C12—C11—H11	120.5
C2—C1—H1	118.8	C8—C12—C11	117.8 (2)
C3—C2—C1	119.3 (2)	C8—C12—H12	121.1
C3—C2—H2	120.4	C11—C12—H12	121.1
C1—C2—H2	120.4	C1—N1—C5	117.71 (18)
C2—C3—C4	118.9 (2)	C1—N1—Ni1	127.46 (15)
C2—C3—H3	120.6	C5—N1—Ni1	114.79 (13)
C4—C3—H3	120.6	C6—N2—N3	113.55 (16)
C5—C4—C3	118.63 (19)	C6—N2—Ni1	113.19 (13)
C5—C4—H4	120.7	N3—N2—Ni1	133.19 (13)
C3—C4—H4	120.7	C7—N3—N2	111.69 (16)
N1—C5—C4	123.14 (19)	C9—N4—C8	116.6 (2)
N1—C5—C6	113.27 (17)	N6—N5—Ni1	119.16 (16)
C4—C5—C6	123.58 (18)	N7—N6—N5	178.8 (2)
N2—C6—C5	120.32 (18)	C6—S1—C7	86.77 (9)
N2—C6—S1	113.49 (15)	N2—Ni1—N2^i^	180.00 (7)
C5—C6—S1	126.18 (15)	N2—Ni1—N1	78.32 (6)
N3—C7—C8	125.80 (19)	N2^i^—Ni1—N1	101.68 (6)
N3—C7—S1	114.47 (15)	N2—Ni1—N1^i^	101.68 (6)
C8—C7—S1	119.72 (15)	N2^i^—Ni1—N1^i^	78.32 (6)
N4—C8—C12	123.7 (2)	N1—Ni1—N1^i^	180.000 (1)
N4—C8—C7	113.74 (19)	N2—Ni1—N5	89.63 (8)
C12—C8—C7	122.5 (2)	N2^i^—Ni1—N5	90.37 (8)
N4—C9—C10	124.4 (3)	N1—Ni1—N5	90.35 (7)
N4—C9—H9	117.8	N1^i^—Ni1—N5	89.65 (7)
C10—C9—H9	117.8	N2—Ni1—N5^i^	90.37 (8)
C9—C10—C11	118.3 (2)	N2^i^—Ni1—N5^i^	89.63 (8)
C9—C10—H10	120.8	N1—Ni1—N5^i^	89.65 (7)
C11—C10—H10	120.8	N1^i^—Ni1—N5^i^	90.35 (7)
C10—C11—C12	119.1 (2)	N5—Ni1—N5^i^	179.998 (1)

Symmetry code: (i) −*x*+2, −*y*+1, −*z*+2.
