# Supplementary figures and images for: Crystal structure of bis­(azido-κN)bis­[2,5-bis­(pyridin-2-yl)-1,3,4-thia­diazole-κ2 N 2,N 3]nickel(II)
Source: Acta Crystallogr E Crystallogr Commun. 2015 Jan 14;71(Pt 2):m24–5. doi: 10.1107/S2056989015000201 (PMC4384553; doi:10.1107/S2056989015000201)

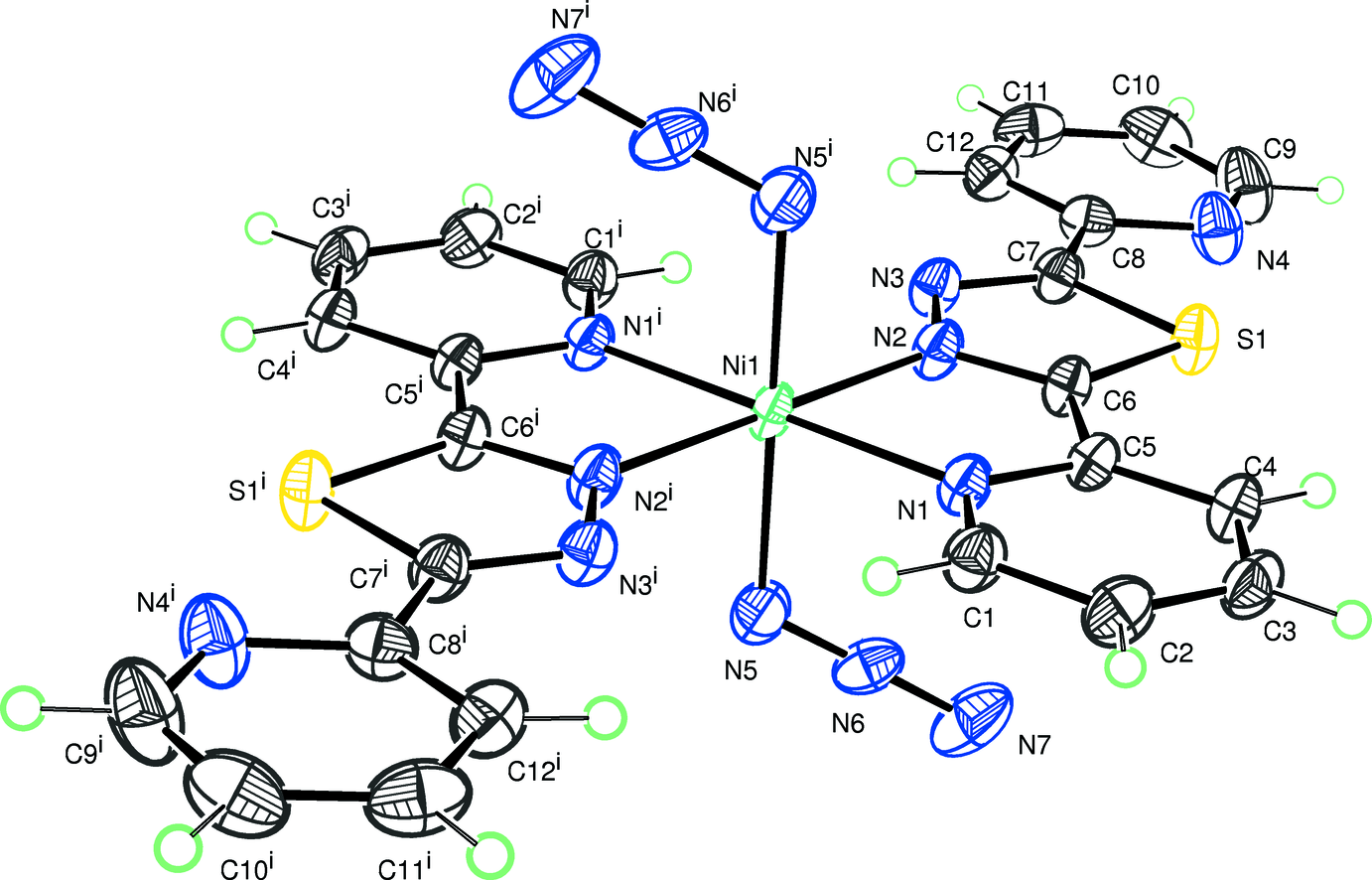

Supplement: Supplementary file 3 [file e-71-00m24-fig1.tif]

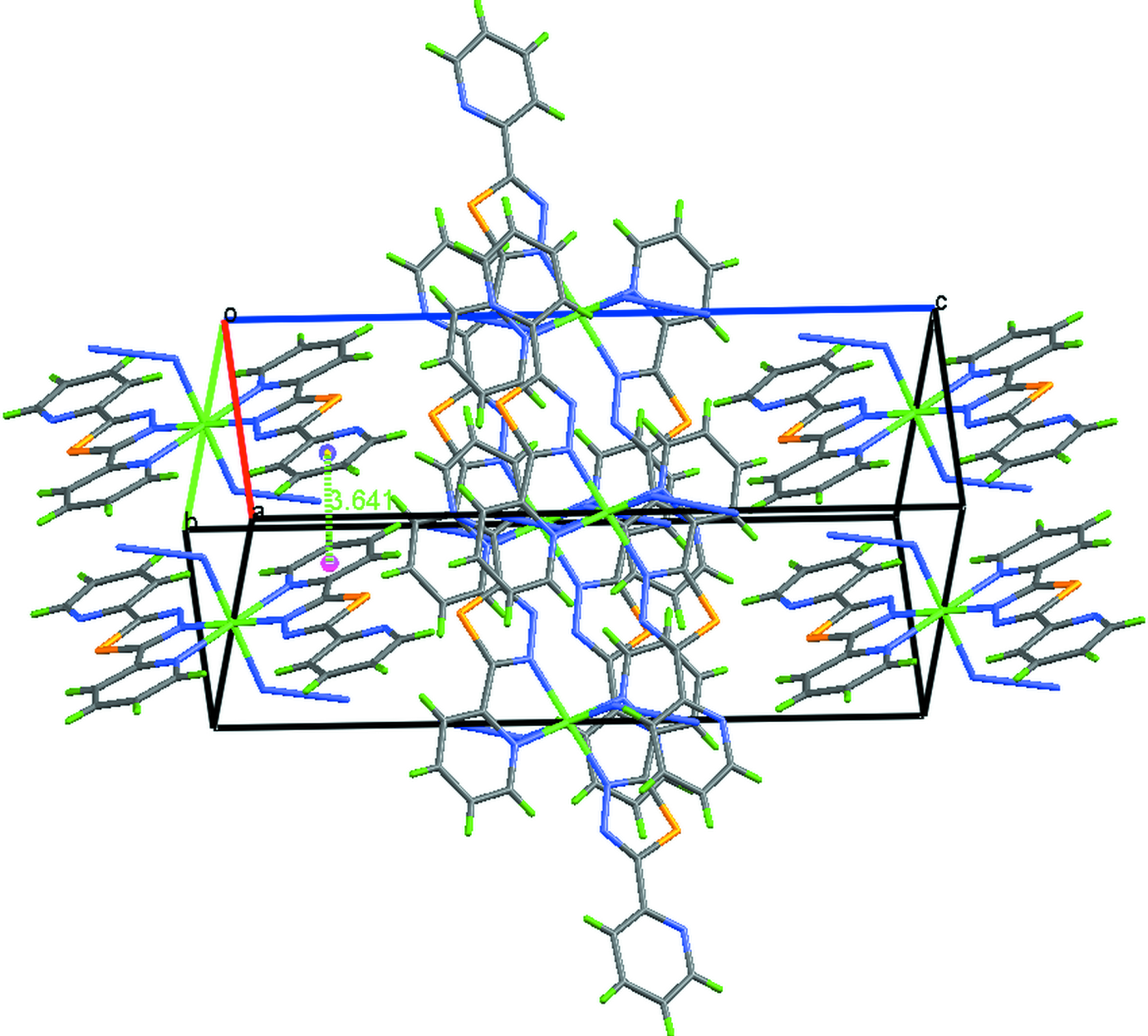

Supplement: Supplementary file 4 [file e-71-00m24-fig2.tif]
